# Using expression data to fine map QTL associated with fertility in dairy cattle

**DOI:** 10.1186/s12711-024-00912-8

**Published:** 2024-06-06

**Authors:** Irene van den Berg, Amanda J. Chamberlain, Iona M. MacLeod, Tuan V. Nguyen, Mike E. Goddard, Ruidong Xiang, Brett Mason, Susanne Meier, Claire V. C. Phyn, Chris R. Burke, Jennie E. Pryce

**Affiliations:** 1 Agriculture Victoria, AgriBio, Centre of AgriBioscience, 5 Ring Road, Bundoora, VIC 3082 Australia; 2 School of Applied Systems Biology, La Trobe University, Bundoora, VIC 3083 Australia; 3 Faculty of Veterinary & Agricultural Science, University of Melbourne, Parkville, VIC 3010 Australia; 4 DairyNZ Limited, Hamilton, 3240 New Zealand

## Abstract

**Background:**

Female fertility is an important trait in dairy cattle. Identifying putative causal variants associated with fertility may help to improve the accuracy of genomic prediction of fertility. Combining expression data (eQTL) of genes, exons, gene splicing and allele specific expression is a promising approach to fine map QTL to get closer to the causal mutations. Another approach is to identify genomic differences between cows selected for high and low fertility and a selection experiment in New Zealand has created exactly this resource. Our objective was to combine multiple types of expression data, fertility traits and allele frequency in high- (POS) and low-fertility (NEG) cows with a genome-wide association study (GWAS) on calving interval in Australian cows to fine-map QTL associated with fertility in both Australia and New Zealand dairy cattle populations.

**Results:**

Variants that were significantly associated with calving interval (CI) were strongly enriched for variants associated with gene, exon, gene splicing and allele-specific expression, indicating that there is substantial overlap between QTL associated with CI and eQTL. We identified 671 genes with significant differential expression between POS and NEG cows, with the largest fold change detected for the *CCDC196* gene on chromosome 10. Our results provide numerous candidate genes associated with female fertility in dairy cattle, including *GYS2* and *TIGAR* on chromosome 5 and *SYT3* and *HSD17B14* on chromosome 18. Multiple QTL regions were located in regions with large numbers of copy number variants (CNV). To identify the causal mutations for these variants, long read sequencing may be useful.

**Conclusions:**

Variants that were significantly associated with CI were highly enriched for eQTL. We detected 671 genes that were differentially expressed between POS and NEG cows. Several QTL detected for CI overlapped with eQTL, providing candidate genes for fertility in dairy cattle.

**Supplementary Information:**

The online version contains supplementary material available at 10.1186/s12711-024-00912-8.

## Background

Female fertility is an important trait in dairy cattle. In the recent past, fertility had declined because of its unfavourable genetic correlation with milk production [1, 2]. The inclusion of fertility in selection indices has now reversed this trend [3, 4]. However, further improvements in fertility traits are desirable [4, 5]. Including sequence variants that are causal variants for fertility traits, or close to them, can help to improve the accuracy of genomic prediction [6, 7]. However, the low heritability and polygenic nature of many fertility traits reduce the power to detect variants that are associated with quantitative trait loci (QTL) [4] and that can be used as prediction markers. Furthermore, to improve prediction accuracy in multiple populations, prediction markers need to be close to the causal mutations [8]. While various QTL have been detected for a range of fertility traits [9–11], identifying causal mutations is more challenging. Recently, Lee et al. [12] combined genome-wide association studies (GWAS) with gene expression data to identify a copy number variant (CNV) on chromosome 6 that is likely the causal mutation underlying a QTL associated with mastitis resistance, calving interval (CI) and milk yield. Similarly, Littlejohn et al. [13] used gene expression data to fine-map a QTL associated with milk production traits, implicating *MGST1* as the causal gene and identifying candidate causal variants for this QTL. Xiang et al. [14] reported an eQTL within the *IRAG1* gene at a site that is conserved across 100 vertebrates and could be a potential causal mutation for birth size via its effect on lipidomics. Hence, using expression data is a promising approach to fine-map QTL and get closer to the causal mutations.

Another approach consists in identifying differences between cows that are genetically divergent for fertility. Meier et al. [15, 16] selected two divergent lines of cows based on their genetic merit for fertility, resulting in a 10-percentage point divergence in the New Zealand fertility breeding value between positive (POS) and negative (NEG) groups. Several studies [15–18] have reported differences in various traits between these two groups, including age at puberty, pregnancy rate, uterine health, interval from calving to ovulation, submission rate, adaptive immune response ranking and peripartum metabolic status. Identifying genetic variants that cause differences between these high and low-fertility cows may aid the identification of causal mutations for fertility. For example, Juengel et al. [19] reported that a missense variant in the *HSD17B12* gene on chromosome 15 was associated with an earlier submission rate for seasonal breeding in these POS and NEG cows.

The breeding objective for fertility in Australia is obtained using a multi-trait model that includes CI, lactation length, calving to first service, pregnancy at the end of the mating season and first-service non-return rate [20]. Among these traits, CI has the largest number of records, with more than 16 million records included in routine genetic evaluations by DataGene (personal communication, Gert Nieuwhof). Hence, CI has the greatest power for a GWAS. Our objective was to combine expression data, fertility phenotypes and allele frequencies in the POS and NEG cows with a GWAS on CI in Australia to fine-map QTL that are associated with fertility in both Australia and New Zealand dairy cattle populations.

## Methods

### Data

**Fig. 1:**
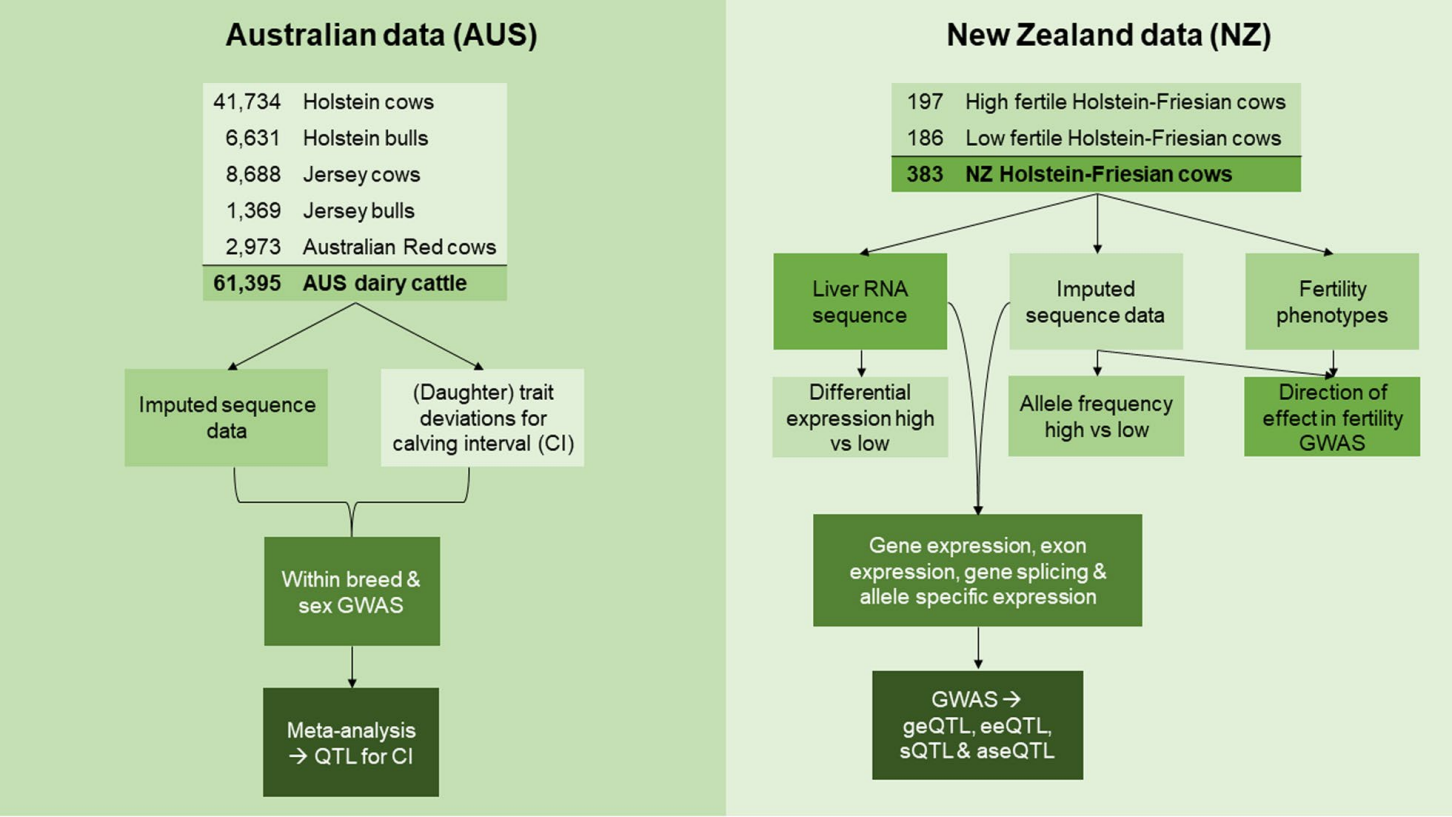
Overview of data used for analyses

We used data from both Australian (AUS) and New Zealand (NZ) dairy cattle. Figure 1 summarises all data sources and analyses. The AUS data included 41,734 Holstein cows, 5631 Holstein bulls, 8688 Jersey cows, 1369 Jersey bulls and 2973 Australian Red cows that had imputed whole-genome sequence and (daughter) trait deviations [21] for CI. The NZ data consisted of 365 Holstein–Friesian cows with imputed whole-genome sequence data, liver biopsies taken on day 7 post-calving, as well as a range of fertility traits measured on heifers and during the first two lactations. Meier et al. [15] described the NZ selection experiment that resulted in cows with a New Zealand fertility breeding value of + 5.0% and − 5.1%, respectively, corresponding to a difference of about 10% in the proportion of cows calved within the first six weeks of the seasonal calving period. A number of fertility traits was measured on these cows. For our analyses, we used traits measured on heifers, and traits measured during the first two lactations. Heifer traits were: age at puberty (agepub, d); submission rate at 3 weeks (sm3wk, binary); submission rate at 6 weeks (sm6wk, binary); pregnancy at 3 weeks (co3wk, binary); pregnancy at 6 weeks (co6wk, binary); time from the planned start of mating to first mating (tstai1, d); and time from planned start of mating to conception (tstconc, d), in a seasonally concentrated natural mating system between October 4 2016 and January 10 2017. Traits measured during the first and second lactation were: co3wk; co6wk; pregnancy at 9 weeks (co9wk, binary); pregnancy at 12 weeks (co12wk, binary); postpartum anovulatory interval determined from twice weekly skim milk progesterone concentrations, using a cut-off of 0.55 µg/mL (ppai, d); ppai censored, where ppai was coded as 1 if ppai was reached by the end of sampling, and 1 otherwise, (ppaicens, binary); final pregnancy at the end of the breeding period (preg, binary); sm3wk; sm6wk; tstai1; tstconc; time from calving to first insemination (ttai1, d); time from calving to conception (ttconc, d); and time from calving to conception censored, coded as 0 for cows that conceived before the end of sampling, and 1 otherwise (conccens, binary). During lactations 1 and 2, cows were managed in a seasonally concentrated breeding system with artificial insemination at 98 and 76 days, respectively. Not all cows had records for all fertility phenotypes (see Additional file 1: Table S1). Cows that were not pregnant after six weeks of mating were synchronised as described by Meier et al. [16]. Based on the New Zealand fertility breeding value and sm6wk, the cows were further split into three groups: 197 cows with a New Zealand fertility breeding value of + 5.0% (POS), 91 cows with a New Zealand fertility breeding value of -5.1% that conceived after six weeks, and 77 cows with a New Zealand fertility breeding value of − 5.1% that did not conceive after six weeks (NEG). All procedures had prior approval from the Ruakura Animal Ethics committee (#13574 & #14200; Hamilton, New Zealand).

### Genotypes

The AUS animals were genotyped with a range of single nucleotide polymorphism (SNP) panels, including various low-density SNP panels, the Illumina Bovine 50K panel (50K) and the Illumina high-density panel (HD). The NZ cows were all genotyped with the GeneSeek Genomic Profiler Bovine 100K BeadChip. Individuals and variants for which the GeneCall score was lower than 0.6 for more than 10% of the genotype calls were discarded, and the remaining genotypes with a GenCall score lower than 0.6 were set to missing. All genotypes were mapped to the ARS-UCD1.2 reference genome [22]. The AUS animals genotyped at a low density were imputed to the 50K panel using the Fimpute v.3 software [23] and a reference population containing 14,722 Holstein, Jersey and Australian Red cattle. Subsequently, the AUS animals were imputed from 50K to HD using Fimpute v.3 [23] with a reference population of 2700 animals, and the NZ animal genotypes were also imputed to the HD panel. Finally, both the AUS and NZ animal HD genotypes were converted to forward sequence format, phased using the Eagle v.2.4.1 software [24], and imputed up to whole-genome sequence with the Minimac 4 software using default settings [25]. The reference population for whole-genome sequence imputation contained 4190 *Bos taurus* cattle that were present in Run8 of the 1000 Bull Genomes Project [26, 27]. After filtering out variants with a Minimac r^2^ < 0.40, a minor allele frequency (MAF) < 0.005 in the AUS and < 0.01 in the NZ animals, 18,247,274 and 14,320,715 sequence variants remained in the AUS and NZ datasets, respectively. For our analyses, we used 13,710,843 variants that overlapped between the AUS and NZ datasets. We used a more stringent MAF filter for the NZ than the AUS data because of the difference in sample size.

### Expression phenotypes

Liver samples used for RNA sequencing were collected from NZ cows at seven days post-calving during first lactation, as described by Grala et al. [18]. Total RNA was extracted from 30 to 150 mg of liver using the TRIzol Plus RNA Purification Kit (Thermo Fisher Scientific) according to the manufacturer’s instructions. Messenger RNA was isolated using NEXTFLEX Poly(A) Beads 2.0 (PerkinElmer) according to the manufacturer’s instructions. Sequencing libraries were prepared using the NEXTFLEX Rapid Directional RNA-Seq Library Prep Kit (PerkinElmer) according to the manufacturer’s instructions and run on a NovaSeq6000 genome analyser (Illumina Inc) in a 150 cycle paired-end run. The read quality of FastQ files was assessed using the FastQC program [28], and FastQ files were trimmed and filtered using the QuadTrim algorithm with a minimum read length of 50, a maximum of five poor-quality bases and a minimum base cut-off quality of 20 [29]. Subsequently, STAR 2-pass mapping [30] was performed for alignment of the RNA reads to the ARS-UCD1.2 reference assembly that is merged with the Btau5 Y chromosome and the Ensembl annotation release 97. The SubRead-featureCounts software [31] was used to obtain gene, exon and intron count matrices, and a hit was called when an overlap was found between a read and a feature. The count matrices were then normalised using TMM normalisation [32] implemented in the edgeR package [33]. Only genes with at least three counts per million reads were retained for further analyses, using the cpm function in edgeR [33]. The LeafCutter software [34] was used to define clusters of introns and generate normalised intron counts per individual for each intron cluster. Introns that were present in less than 60% of the individuals and those that showed almost no variation were discarded. To obtain allele ratio counts for allele-specific expression analyses, we used the GATK software [35] to first generate a reference dictionary, followed by GATK SplitNCigarReads to enable the recognition of split reads. The VCF files were then generated using the GATK Haplotype Caller and GATK GenotypeGVCFs. From the VCF files, we generated allele counts for the two most prevalent alleles, filtering out sites with a sum of allele counts less than 10, sites with a difference between read depth and sum of allele counts greater than 20%, and those for which the two most prevalent alleles did not match the reference and alternate alleles. The allele count phenotype that was used for downstream analyses was calculated as log $\left(\frac{nRef+10}{nAlt+10}\right)$, where $nRef$ is the allele count of the reference allele, and $nAlt$ is the allele count of the alternative allele.

### GWAS for fertility traits

GWAS were performed for CI in the AUS dataset and for fertility traits in the NZ dataset. For the AUS dataset, first a within-breed and -sex GWAS was undertaken using the mixed linear model association (MLMA) analyses in GCTA [36], by fitting a genomic relationship matrix (GRM) based on HD genotypes. The GRM were constructed following Yang et al. [37]. Subsequently, the five AUS within-breed/sex GWAS were combined in a multi-breed meta-analysis (AUS_CI) using the weighted Z-score model as implemented in the METAL software [38]. All GWAS on the NZ data were performed using GCTA [36]. We considered that all the variants with a p-value ≤ 10^–6^ in the AUS meta-analysis were significantly associated with CI. The corresponding false discovery rate (FDR) was estimated as ${Q}_{e}=E\left(Q\right)=E\left\{V/(V+S)\right\}=E(V/R)$ [39], where ${Q}_{e}$ is the expectation of the proportion of false positives, $V$ is the number of true null hypotheses that are declared significant (number of false positives), $S$ is the number of non-true null hypotheses that are declared significant (number of true positives), and $R=V+S$. With a threshold of 10^–6^, the expectation of $V$ equals the total number of tests × 10^–6^. To define QTL regions, first we ranked all the significant variants from the smallest to largest p-value, and then selected the most significant variants, grouping the variants that were within 1 Mb of a most significant variant as part of that QTL region. For the NZ fertility traits, we used the leave-one-chromosome-out approach in GCTA [36], to maximise the power of the GWAS on the smaller NZ dataset. The GRM included in the model was constructed following Yang et al. [37], using HD genotypes. Fixed effects and covariates fitted for the NZ fertility traits were calving age, calving month, and, for the traits measured after six weeks of mating, synchronisation. We made use of only the direction of the effect obtained from the GWAS for the NZ fertility traits and did not attempt to detect QTL because of the low power due to the small number of NZ animals recorded.

### Differential expression analysis

We used the normalised gene counts and the exactTest function in edgeR [33] to detect genes that were differentially expressed between the POS and NEG cows. Genes with a FDR < 0.05 were declared significant.

### GWAS for expression phenotypes

GWAS were performed on the gene counts, exon counts, intron counts, and allele counts to detect gene expression QTL (geQTL), exon expression QTL (eeQTL), splicing QTL (sQTL) and allele specific expression QTL (aseQTL), respectively. The gene counts, exon counts, and intron counts were fitted as phenotypes in GCTA [36], with age fitted as a covariate and a GRM based on the HD genotypes fitted as a random effect. Only variants that were within 1 Mb of a gene, exon or intron were included in these GWAS. The detection of geQTL, eeQTL and sQTL was performed as described for the detection of QTL for CI. To detect allele-specific expression QTL (aseQTL), we tested the association between the driver SNP (dSNP) and the transcript SNP (tSNP), where the tSNP was the variant for which we estimated the allele count phenotypes, and the dSNPs were all the variants that were within 1 Mb of the tSNP. We performed two association tests for each combination of tSNP and dSNP [40], using inhouse scripts. Both tests used only samples that were heterozygous at both the tSNP and dSNP. Because this reduced the sample size, we used a MAF threshold of 0.05 for the allele-specific expression (ASE) tests. The first ASE test was a linear model:

1 $$\mathbf{y}=\mathbf{x}\mathbf{b}+\mathbf{e},$$

where $\mathbf{y}$ is a vector of the allele count phenotypes of the tSNP, $\mathbf{x}$ is a vector of the genotypes at the dSNP coded as 1 if the dSNP had the same phase as the tSNP and − 1 if the dSNP had an alternate phase, $\mathbf{b}$ is a vector of the effects of dSNP on tSNP, and $\mathbf{e}$ is a vector of random residuals. This linear model can lead to many false positives when the sample has a limited size. Therefore, we also performed a second, more stringent ASE test to filter cases for which the sample size was too small for the linear model test. The second ASE test was a Z-test, for which we first calculated:

- ${M}_{i}$ = the number of counts of the reference allele at the tSNP for each individual $i$ with the dSNP and tSNP genotypes having the same phase;
- ${P}_{i}$ = the number of counts of the alternate allele at the tSNP for each individual $i$ with the dSNP and tSNP genotype having the same phase;
- ${N}_{j}$ = the number of counts of the reference allele at the tSNP for each individual $j$ with the dSNP and tSNP genotype having alternate phases;
- ${Q}_{j}$ = the number of counts of the alternate allele at the tSNP for each individual $i$ with the the dSNP and tSNP genotypes having alternate phases.

Subsequently, the Z-score was calculated as:

2 $$Z=\left(\frac{A}{T}-0.5\right)\times \sqrt{4T},$$

where $A=\sum {M}_{i}+\sum {Q}_{j}$ and $T=\sum {M}_{i}+\sum {P}_{i}+\sum {N}_{j}+\sum {Q}_{j}$. The detection of aseQTL was done as described for CI, except that a variant had to have p-values ≤ 10^–6^ for both ASE tests to be declared significant, in order to have the aseQTL detection with the highest confidence.

### Enrichment analyses

To estimate the enrichment of eQTL, first we calculated the percentage of all the variants that were eQTL ($${perc}_{eQTL\_all}$$), for each type of eQTL (geQTL, eeQTL, sQTL, and aseQTL). Subsequently, we calculated the percentage of variants that were eQTL for those that were significantly associated with CI ($${perc}_{eQTL\_CI}$$). The enrichment was then quantified as $${perc}_{eQTL\_CI}/{perc}_{eQTL\_all}$$$$.$$ We tested if the observed enrichment was significant using a chi-square test, using the chisq.test() function in R [41]. The enrichment analyses were performed using significance thresholds of 10^–4^, 10^–6^ and 10^–8^, to test how sensitive the enrichment was to the significance threshold.

### Comparison of NZ fertility traits and AUS CI

To assess the link between CI in AUS cows and NZ fertility traits, we estimated the percentage of variants for which the direction of effect for each of the NZ fertility traits was consistent with that of AUS CI, e.g., the allele that decreases CI in AUS improves the fertility trait in NZ, and the allele the increases CI in AUS reduces fertility in NZ. This was estimated for all the variants included in the analyses, for the variants that were significantly associated with CI, and for the variants that were significantly associated with both CI and gene expression, exon expression, gene splicing or allele specific expression.

### Overlap between QTL for CI and eQTL

We considered that the QTL detected for different phenotypes (e.g., QTL detected for CI and QTL detected for expression phenotypes) overlapped if variants were significant for both phenotypes. For those variants, we assumed that a variant was more likely to be the causal variant underlying the QTL and eQTL, if the direction of the effect was concordant for all the estimates (see Additional file 2: Fig. S1). E.g., if a QTL for CI overlapped with a geQTL, and if the allele that decreased CI increased gene expression, then we expected that, in the differential expression analyses, this gene would be upregulated in the high-fertility cows, and would have a favourable effect on the GWAS NZ fertility traits. Furthermore, we expected that this allele would have a higher allele frequency in the POS than the NEG cows.

## Results

### RNA sequencing and enrichment analyses

The RNA sequencing pipeline resulted in an average of 24,513,204 read pairs per sample, of which 89.32% were uniquely aligned (see Additional file 3: Table S2). The number of variants analysed and the FDR for the detection of variants associated with gene expression (GE), exon expression (EE), gene splicing (S) and ASE using significance thresholds of 10^–6^ are in Table 1. In total, 3412 variants were significantly associated with CI, corresponding to an FDR of 4.0 × 10^–3^. The variants that were significantly associated with CI were highly enriched for variants associated with all four of the expression phenotypes (Table 1). For example, while 7.43% of the variants analysed were significantly associated with the expression of at least one exon, 62.05% of these variants were also significantly associated with CI, corresponding to an enrichment fold of 8.3 (p_enrichment_ ≈ 0). Enrichment folds were sensitive to the significance thresholds used (see Additional file 4: Table S3). Enrichment for geQTL and eeQTL was highest with the more stringent significance thresholds (up to 8.6 and 15.6 fold enrichment, for geQTL and eeQTL, respectively), and was highly significant regardless of all the thresholds tested. Enrichment for sQTL was less sensitive to the significance thresholds (between 1.3 and 2.2 fold enrichment), with a slightly higher level of enrichment using a less stringent significance threshold in the GWAS for CI. Enrichment for aseQTL varied widely with the different significance thresholds. For example, using a significance threshold of 10^–8^ in the GWAS for CI, a threshold of 10^–6^ to detect aseQTL resulted in an enrichment factor of 11.5 (p ≈ 0), but with a more stringent threshold of 10^–8^, there was no overlap between aseQTL and QTL associated with CI anymore, resulting in significant (p = 2.2 × 10^–6^) depletion.

**Table 1:** Enrichment of variants associated with calving interval (CI) for eQTL

Set	n_total_	perc_eQTL_all_	FDR	n_signCI_	perc_eQTL_CI_	Enrichment	p_enrichment_
All	13,710,843	–	–	3412	0.02^a^	–	–
GE	1,196,131	8.72^a^	7.5 × 10^–5^	1719	50.38^b^	5.8	0
EE	1,019,185	7.43^a^	1.4 × 10^–4^	2117	62.05^b^	8.3	0
S	2,019,663	14.73^a^	2.5 × 10^–4^	813	23.83^b^	1.4	4.5 × 10^–26^
ASE	305,494	2.23^a^	4.4 × 10^–4^	604	17.70^b^	7.9	0

*FDR *GWAS false discovery rate, *n*_*total*_ total number of variants included in set, *n*_*signCI*_ number of variants significantly associated with calving interval (CI), *GE* variants significantly associated with gene expression, *EE* variants significantly associated with exon expression, *S* variants significantly associated with gene splicing, *ASE* variants significantly associated with allele specific expression, expressed in % of the 13,7 M variants included in the GWAS for CI^a^, or the 3412 variants significantly associated with CI^b^, enrichment = perc_eQTL_CI_/perc_eQTL_all_, p_enrichment_ = p-value of the Chi-square test enrichment

### Comparison of QTL and eQTL with New Zealand fertility traits

The percentage of the variants with the expected direction of effect on both CI in AUS and the NZ fertility traits measured during lactation 1 (${\%dir}_{AUS,NZ}$) ranged from 57% (for co9wk) to 82% (for ppaicens) for variants that were significantly associated with CI (Table 2). When the variants were associated with both CI and either GE, EE or ASE, %*dir*_*AUS*,*NZ*_ increased up to 94, 96 and 99%, respectively. However, for all traits, %*dir*_*AUS*,*NZ*_ was lower for the variants that were associated with both CI and gene splicing than for variants that were only associated with CI, with %*dir*_*AUS*,*NZ*_ ranging from 25 to 75%. New Zealand fertility traits measured on heifers and during lactation 2 had %*dir*_*AUS*,*NZ*_ < 50% for several traits, with %*dir*_*AUS*,*NZ*_ of the variants that were associated with CI ranging from 27 to 79% for the traits measured on heifers, and from 30 to 85% for those measured during lactation 2 (see Additional file 5: Table S4).

**Table 2:** Percentage of variants with a direction of the effect on fertility consistent between the Australian and New Zealand datasets

Trait	Variants					
	ALL	CI	CI + GE	CI + EE	CI + SPLICE	CI + ASE
co3wk	51	80	94	96	75	99
co6wk	51	76	87	89	57	98
co9wk	51	57	77	69	51	90
co12wk	51	70	89	91	69	96
ppai	50	78	89	88	63	94
ppaicens	50	82	79	83	56	94
preg	51	70	89	91	69	96
sm3wk	50	79	76	82	41	98
sm6wk	50	65	73	80	25	98
tstai1	50	78	76	82	43	98
tstconc	51	71	88	91	62	97
ttai1	50	80	81	83	51	94
ttconc	51	71	92	94	69	95
conccens	51	70	89	91	69	96

Trait = New Zealand fertility trait measured during lactation 1, co3wk/co6wk/co9wk/co12wk = pregnancy rate at 3/6/9/12 weeks, ppai = prolonged postpartum anovulatory intervals, ppaicens = ppai censored, preg = pregnancy rate, sm3wk/sm6wk = submission rate at 3/6 weeks, tstai1 = time from planned start of mating to 1st AI, tstconc = time from planned start of mating to conception, ttai1 = time from calving to 1st AI, ttconc = time to conception, concsens = ttconc censored, ALL = all variants included in the study, CI = variants that were significantly associated (p ≤ 10^–6^) with calving interval in Australia, CI + GE/CI + EE/CI + SPLICE/CI + ASE = variants that were significantly associated (p ≤ 10^–6^) with calving interval in Australia and gene expression/exon expression/gene splicing/allele specific expression in New Zealand

### Differential expression

**Fig. 2:**
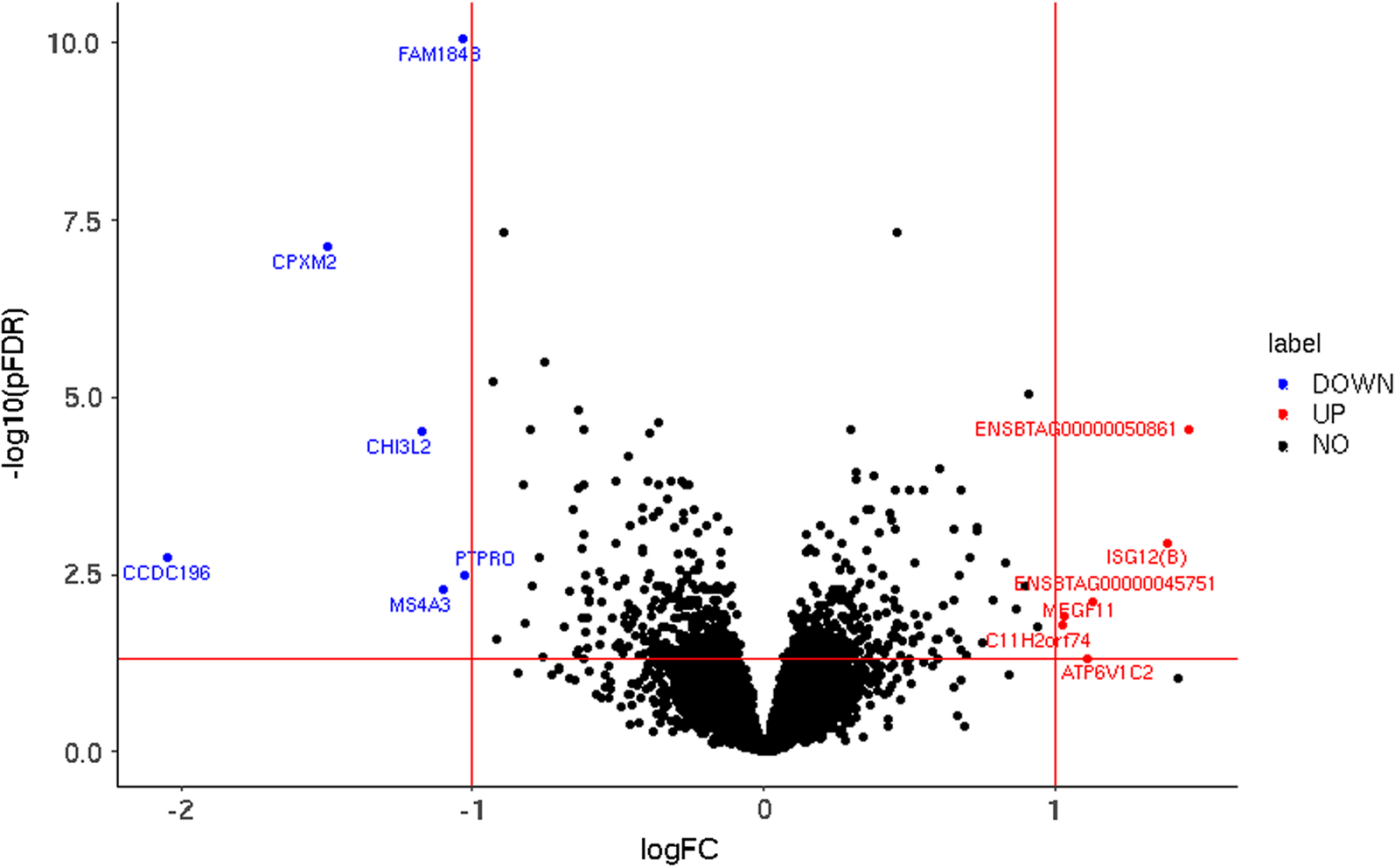
Volcano plot of differentially expressed genes. ‘Down’ indicates variants with a FDR ≤ 0.05 and a log2 fold change (logFC) smaller than 1, ‘up’ indicates variants with a FDR ≤ 0.05 and a log2 fold change (logFC) larger than 1, ‘no’ indicates all other variants

Six hundred and seventy-one genes displayed a significant differential expression between the POS and NEG NZ cows (see Additional file 6: Table S5) and among these, 295 and 376 were, respectively, upregulated and downregulated in the POS fertile cows. Eleven genes had a log2 fold change ≥ 1 (Table 3, Fig. 2). While none of the top variants that were associated with the expression of these genes was significantly associated with CI, for eight of these 11 genes the direction of the effect on CI was consistent with the expectation based on the fold change and effect on gene expression. For example, the *coiled-coil domain containing 196* (*CCDC196*) gene on chromosome 10 was downregulated in POS NZ cows. Therefore, the allele that increases expression of *CCDC196* was expected to increase CI, which was the case. The only genes for which we found that the direction of effect on CI was not consistent with the expectation based on the fold change and effect on gene expression were: *carboxypeptidase X, M14 family member 2* (*CPXM2*) on chromosome 26, *ENSBTAG00000050861* on chromosome 4, and *chitinase 3 like 2* (*CHI3L2*) on chromosome 3. Of all the 671 significantly differentially expressed genes, 322 had a top variant for which the direction of effect on gene expression and CI were in line with the direction of the fold change.

**Table 3:** Differentially expressed genes with the largest fold change

Gene	Chr	Start	End	logFC	NEG	POS	FDR	topGE	Allele	pGE	dirGE	pCI	dirCI
*CCDC196*	10	78,535,568	78,551,038	− 2.04	22	5	1.9 × 10^–3^	79,213,632	T	8.0 × 10^–68^	+	0.18	+
*CPXM2*	26	43,495,815	43,632,754	− 1.50	121	42	7.7 × 10^–8^	43,153,598	G	1.9 × 10^–10^	+	0.44	–
*ENSBTAG00000050861*	4	112,184,180	112,196,007	1.46	17	34	2.8 × 10^–5^	111,932,109	T	4.5 × 10^–5^	−	0.12	−
*ISG12(B)*	21	58,715,675	58,727,005	1.38	18	34	1.2 × 10^–3^	58,743,545	T	4.1 × 10^–6^	+	0.35	−
*CHI3L2*	3	32,155,439	32,192,735	− 1.17	85	37	3.1 × 10^–5^	32,180,704	C	2.8 × 10^–6^	−	0.66	+
*ATP6V1C2*	11	86,882,894	86,942,882	1.11	13	27	4.8 × 10^–2^	87,272,584	A	2.9 × 10^–3^	−	0.15	+
*MS4A3*	15	83,149,879	83,159,312	− 1.10	10	16	5.2 × 10^–3^	83,092,648	C	5.1 × 10^–7^	+	0.36	+
*FAM184B*	6	37,181,075	37,301,540	− 1.03	7	3	8.8 × 10^–11^	36,628,669	T	1.5 × 10^–6^	−	0.63	−
*MEGF11*	10	12,771,484	13,163,546	1.03	36	14	1.2 × 10^–2^	12,106,041	C	2.7 × 10^–32^	+	0.50	−
*PTPRO*	5	94,342,435	94,455,619	− 1.03	6	10	3.2 × 10^–3^	93,515,249	C	4.5 × 10^–10^	+	0.99	+
*C11H2orf74*	11	59,794,837	59,822,193	1.02	4	2	1.7 × 10^–2^	59,666,616	T	3.3 × 10^–39^	+	0.60	–

*Gene* gene symbol, *Chr* chromosome, *start* start of gene in base pair (bp), *end* end of gene in bp, *logFC* logarithm of fold change, positive value means the gene was upregulated in the high fertile cows compared to the low fertile cows, *NEG* average expression in the very low fertile cows, *POS *average expression in the high fertile cows, *topGE* most significant variant in the gene expression GWAS (if there were more than one variant with the smallest p-value, we selected the variant that had the smallest p-value in the meta-analysis for calving interval), *Allele *allele for which GWAS results are reported, *pGE* p-value in the gene expression GWAS, *dirGE* direction of effect of allele on gene expression, *pCI* p-value in the meta-analysis for calving interval, *dirCI* direction of effect of allele on calving interval

### Overlap between QTL for CI and eQTL

**Fig. 3:**
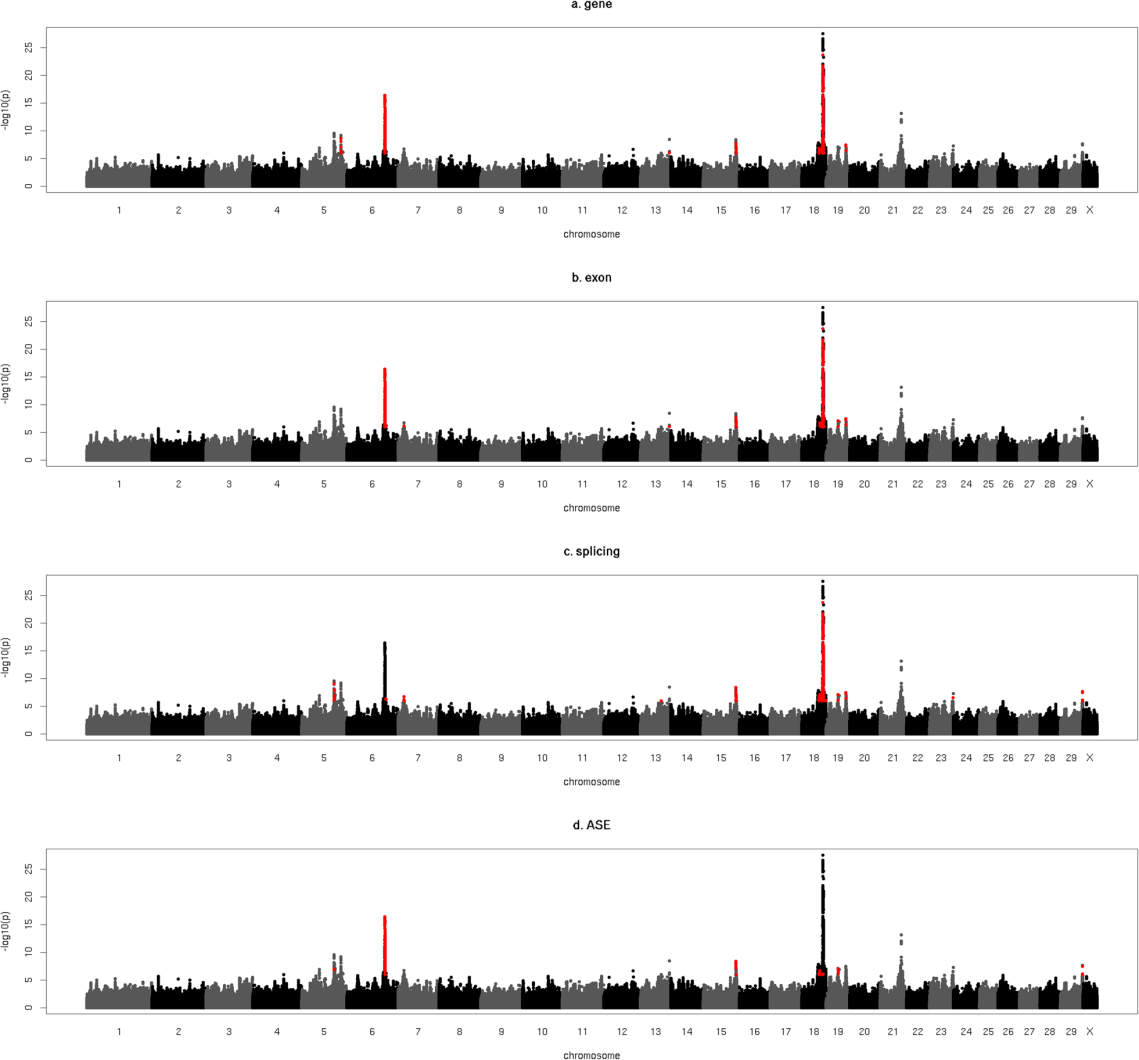
Overlap between QTL for calving interval and eQTL. Y-axis = − log10(p) for the meta-analysis of calving interval (CI), red indicates variants that are significant (p ≤ 10^–6^) for both CI and (**a**) gene expression (gene), (**b**) exon expression (exon), (**c**) gene splicing (splicing) and (**d**) allele specific expression (ASE)

Figure 3 visualises all the overlaps between QTL for CI and geQTL, eeQTL, sQTL and aseQTL. Overlaps were detected on chromosomes 5, 6, 7, 13, 15, 18, 19, 23 and 29. We provide more details for five QTL regions that are located on chromosomes 5, 6, 15 and 18 (see Table 4) and include the two most significant QTL detected for CI that overlap with eQTL.

**Table 4:** Examples of QTL regions detected for calving interval that contain eQTL

Chr	Start	End	nCI	Freq_high	Freq_veryLow	Top	p	nGE	nEE	nSplice	nASE
5	88,012,951	90,491,717	58	0.74 ± 0.21	0.76 ± 0.25	88,448,176	2.7 × 10^–10^	0	0	9	0
5	105,374,211	106,005,410	82	0.25 ± 0.14	0.26 ± 0.13	105,874,904	6.4 × 10^–10^	6	0	0	0
6	86,594,473	87,937,448	1667	0.54 ± 0.13	0.50 ± 0.13	87,070,486	3.5 × 10^–17^	1133	1658	0	528
15	76,536,678	77,769,100	38	0.23 ± 0.14	0.19 ± 0.16	76,536,678	3.8 × 10^–9^	9	38	18	26
18	55,331,602	60,178,330	1310	0.77 ± 0.18	0.78 ± 0.16	57,689,072	3.2 × 10^–35^	545	429	690	0

*Chr* chromosome, *start* start of QTL region in base pair (bp), *end* end of QTL region in bp, *nCI* number of variants in QTL region associated with calving interval (CI), *Freq_high* average allele frequency of alleles that increase CI in high fertile cows, *Freq_veryLow* average allele frequency of alleles that increase CI in very low fertile cows, *top* location of the most significant variants in the QTL region, *p* p-value of the top variant for the association with CI, nGE/nEE/nSplice/nASE, number of variants associated both with CI and gene expression/exon expression/gene splicing/allele specific expression

**Fig. 4:**
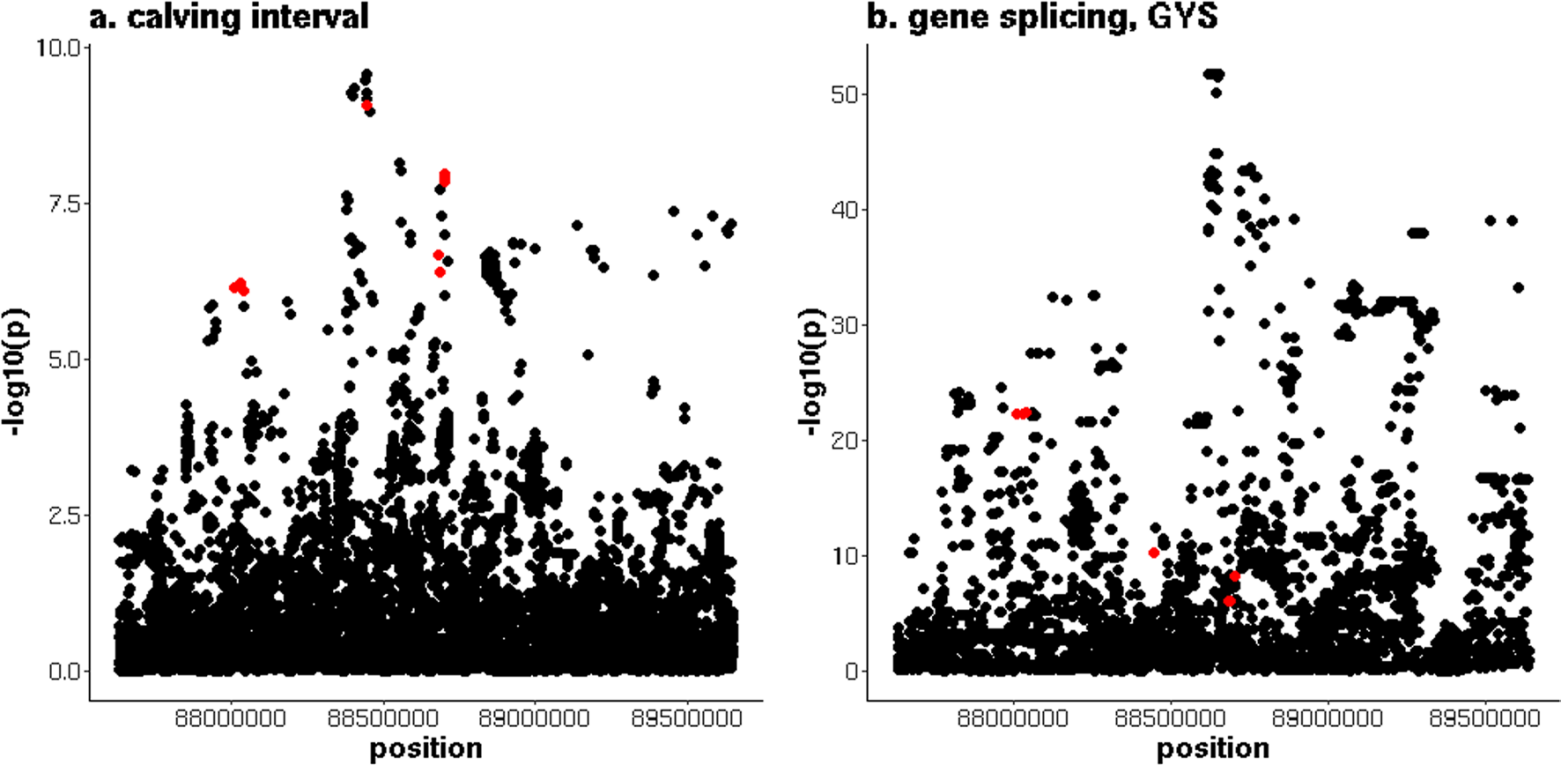
Overlap between a QTL for calving interval on chromosome 5 and *GYS2* splicing. **a** y-axis = − log10(p) for the meta-analysis of calving interval (CI), and (**b**) y-axis = − log10(p) for the intron in *GYS2* located between 88,636,100 and 88,647,589 bp, red indicates variants that are significant (p ≤ 10^–6^) for both CI and gene splicing

**Fig. 5:**
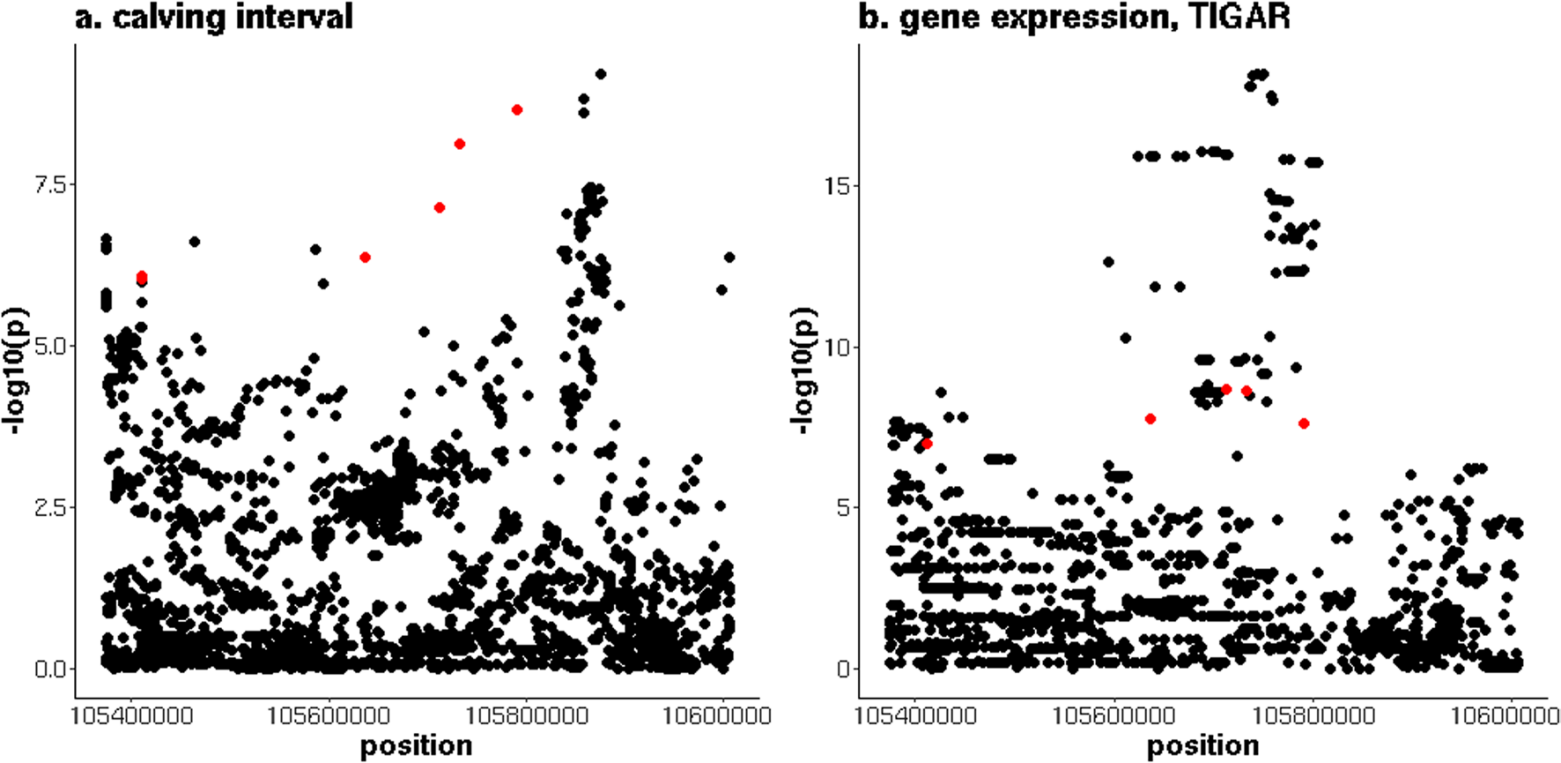
Overlap between a QTL for calving interval on chromosome 5 and *TIGAR* expression. **a** y-axis = − log10(p) for the meta-analysis of calving interval (CI), and (**b**) y-axis = − log10(p) for *TIGAR* expression, red indicates variants that are significant (p ≤ 10^–6^) for both CI and gene expression

On chromosome 5, there were two distinct peaks associated with CI. The QTL located between 88,012,951 and 90,491,717 bp, contained 58 variants that were associated with CI in the AUS data. Ten variants were associated with CI and alternative splicing of the *glycogen synthase 2* (*GYS2*) and *pyridine nucleotide-disulphide oxidoreductase domain 1* (*PYROXD1*) genes. The most significant sQTL (p = 4.5 × 10^–23^) was associated with an intron of the *GYS2* gene that is located between 88,636,100 and 88,647,589 bp and for which nine variants overlapped with the QTL for CI (Fig. 4 and see Additional file 7: Table S6). Two of these nine variants had a direction of effect on CI that was consistent with the allele frequencies in the POS and NEG cows, and the direction of effect on the first lactation traits in the NZ cows. These two variants were located at 88,702,039 and 88,702,059 bp, in the *RecQ like helicase* (*RECQL*) gene and were in complete linkage disequilibrium (LD) in the NZ dataset. In addition, the alleles that increased CI had an allele frequency of 0.79 and 0.88 in the POS and NEG cows, respectively, and reduced fertility for all first lactation traits. The second QTL on chromosome 5 was located between 105,374,211 and 106,005,410 bp, with 82 variants significantly associated with CI. In the same region, there were 287 variants associated with the expression of the *tumor protein 53 induced glycolysis regulatory phosphatase* (*TIGAR*) gene. Six variants were associated with both CI and *TIGAR* expression (Fig. 5 and see Additional file 7: Table S6), with minimum p-values for CI and *TIGAR* expression of 2.2 × 10^–9^ and 2.1 × 10^–9^, respectively. For these six variants, the alleles that increased CI reduced the expression of *TIGAR*. This agrees with the positive fold change of *TIGAR* expression in the differential expression analyses (not significant, p = 0.05, FDR = 0.21). For one of the six variants, the allele that increased CI and reduced *TIGAR* expression, was more common in the NEG (allele frequency = 0.53) than in the POS (allele frequency = 0.35) NZ cows and had an unfavourable effect on all first lactation traits. This variant was located at 105,732,916 bp, in the 3’UTR region of *TIGAR*.

**Fig. 6:**
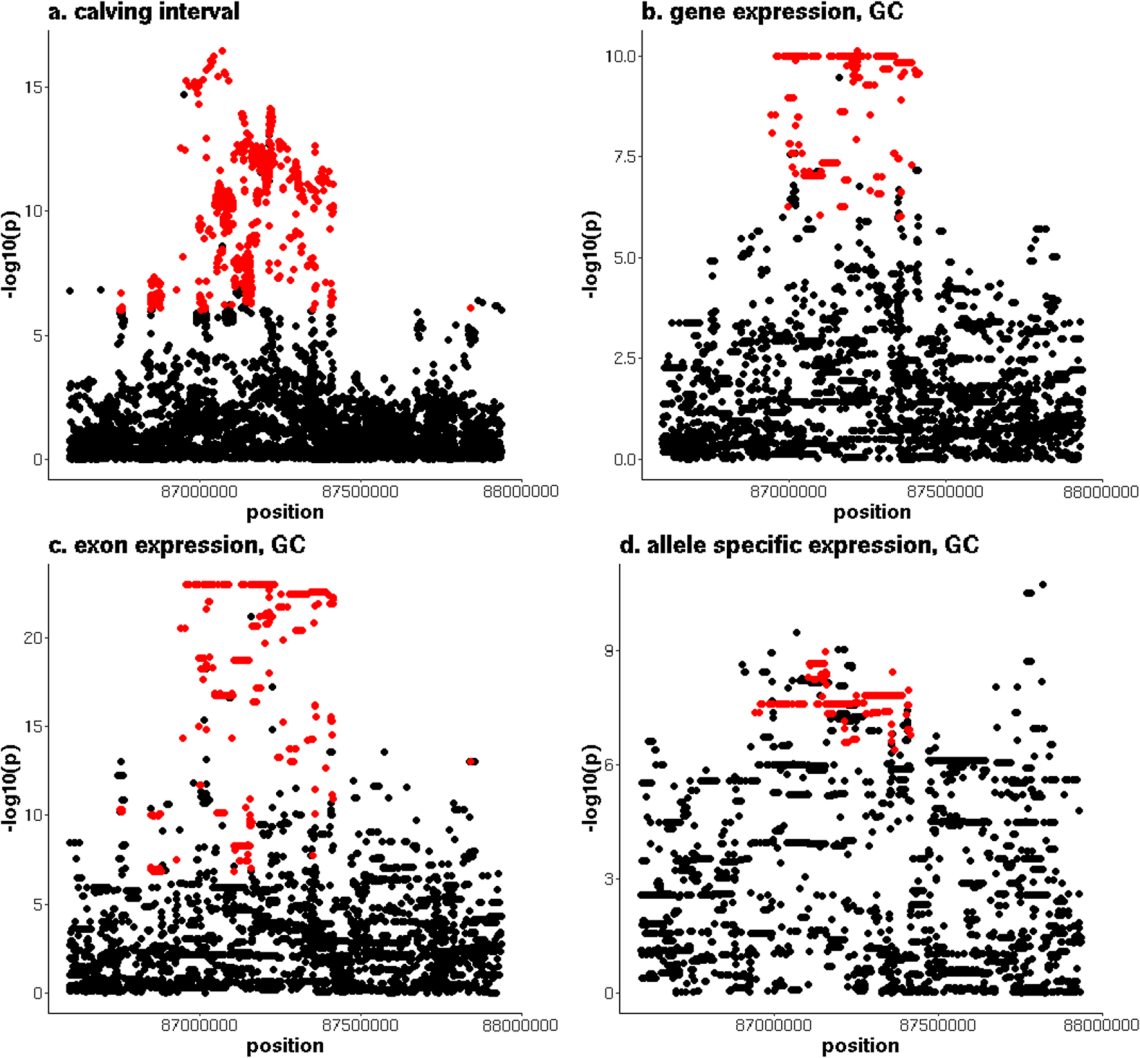
Overlap between a QTL for calving interval on chromosome 6 and multiple types of eQTL. **a** y-axis = − log10(p) for the meta-analysis of calving interval (CI), (**b**) y-axis = − log10(p) for *GC* expression, (**c**) y-axis = − log10(p) for exon expression of exon of *GC* located between 86,968,870 and 86,968,921 bp, and (**d**) y-axis = − log10(p) for allele expression of tSNP located at 86,989,953 bp, red indicates variants that are significant (p ≤ 10^–6^) for both CI and expression phenotype

The second most significant QTL detected for CI (p = 3.5 × 10^–17^) was located between 86,594,473 and 87,937,448 bp on chromosome 6. Of the 1667 variants that were significantly associated with CI, 1133, 1658 and 528 were significantly associated with expression of the *GC vitamin D binding protein* (*GC*) gene (minimum p-value = 7.7 × 10^–11^), exon expression of multiple exons in *GC* (minimum p-value = 1.1 × 10^–23^), and ASE within *GC* (minimum p-value = 1.5 × 10^–8^), respectively (Fig. 6 and see Additional file 7: Table S6). All the 1133 alleles associated with an increased expression of *GC* were associated with increased CI, and *GC* was downregulated in the POS cows (not significantly, p = 0.07, FDR = 0.24). However, there were only five variants for which the allele that increased *GC* expression was more common in the NEG cows than in the POS cows and had an unfavourable association with the first lactation traits. These variants included an intronic variant of *GC* (86,996,470 bp), three intergenic variants (87,020,854, 87,038,423 and 87,099,129 bp) and an intronic variant of the *neuropeptide FF receptor 2* (*NPFFR2*) gene at 87,257,944 bp. The most significant eeQTL was associated with an exon of *GC*, located between 86,968,870 and 86,968,921 bp. In total, 1634 variants were significantly associated with this exon, and for all of these variants, the allele that was associated with an increased expression of this exon, increased CI. For 88 of these variants, the allele frequency and direction of effect on the first lactation traits in NZ cows were consistent with the direction of effect on CI in AUS cows. The largest difference in allele frequency was found for an intergenic variant located at 87,020,854 bp, where the allele that was associated with increased CI had an allele frequency of 0.54 in the NEG cows and 0.45 in the POS cows. There was one tSNP for which significant dSNPs overlapped with significant variants for CI, and which was a synonymous variant of *GC* located at 86,989,953 bp. Five hundred and twenty-eight dSNPs were significantly associated with ASE and CI, including 14 variants for which the allele that was associated with increased CI was more common in the NEG cows than in the POS cows. For these 14 variants, the direction of effect was not consistent for all first lactation traits in NZ cows.

**Fig. 7:**
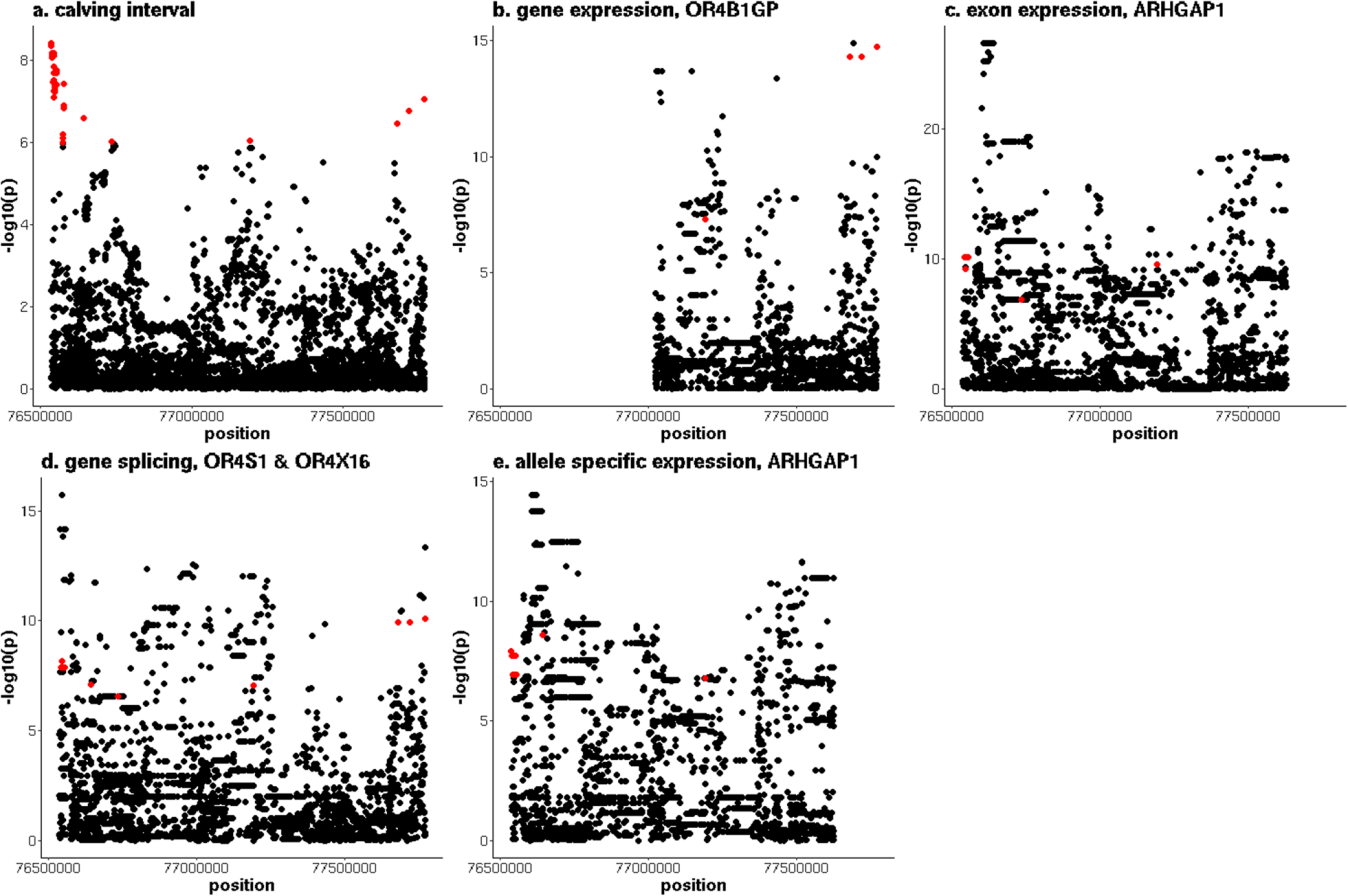
Overlap between a QTL for calving interval on chromosome 15 and multiple types of eQTL. **a** y-axis = − log10(p) for the meta-analysis of calving interval (CI), **b** y-axis = − log10(p) for *OR4B1GP* expression, **c** y-axis = − log10(p) for exon expression of exon of *ARHGAP1* located between 76,627,377 and 76,627,508 bp, **d** y-axis = − log10(p) for the splice region between 77,959,141 and 77,997,756 bp, and (**e**) y-axis = − log10(p) for allele expression of tSNP located at 76,623,841 bp, red indicates variants that are significant (p ≤ 10^–6^) for both CI and expression phenotype

A QTL region located between 76,536,678 and 77,769,100 bp on chromosome 15 overlapped with multiple geQTL, eeQTL, sQTL and aseQTL (Fig. 7 and see Additional file 7: Table S6). The most significant geQTL (p = 1.8 × 10^–15^) was associated with the expression of the *olfactory receptor family 4 subfamily B member 1G, pseudo* (*OR4B1GP*) gene. In total, nine variants were significantly associated with CI and geQTL, including five variants that were associated with the expression of the *Rho GTPase activating protein 1* (*ARHGAP1*) and *CAMP responsive element binding protein 3 like 1* (*CREB3L1*) genes, and three variants associated with the expression of the *olfactory receptor family 4 subfamily X member 17* (*OR4X17*), *olfactory receptor family 4 subfamily A member 47V* (*OR4A47V*) and *OR4B1GP* genes. None of these genes were significantly differentially expressed between the POS and NEG NZ cows. However, the direction of the fold change was consistent with the estimated directions of the effect on CI and gene expression for the *ARHGAP1* and *OR4A47V* (upregulated in the POS cows, and the alleles that were associated with increased gene expression were associated with reduced CI) and *CREB3L1* genes (downregulated in the POS cows, and alleles that were associated with increased gene expression were associated with increased CI). However, this was not the case for the *OR4B1GP* and *OR4X17* genes (upregulated in POS cows, but the alleles that were associated with increased gene expression were associated with increased CI). Allele frequencies in the POS and NEG cows were not in agreement with the direction of the estimated effects on CI and gene expression. Significant eeQTL were detected for exons of the *CREB3L1*, *ARHGAP1* and *OR4X17* genes, with the most significant eeQTL (p = 7.7 × 10^–11^) detected for an exon of *ARHGAP1*, located between 76,627,377 and 76,627,508 bp. The most significant sQTL (p = 8.2 × 10^–11^) in the region was associated with a splice region between 77,959,141 and 77,997,756 bp located in a copy number variant (CNV) that encompassed seven genes of the olfactory receptor family 4 (*subfamily B member 1F* (*OR4B1F*), *olfactory receptor family 4 subfamily X member 5* (*OR4X5*), *OR4X17*, *olfactory receptor family 4 subfamily X member 16* (*OR4X16*) and *OR4B1GP*) [42]. All the significant ASE variants that overlapped with the QTL for CI were associated with a tSNP that is located in the 3’UTR region of *ARHGAP1* at 76,623,841 bp (minimum p-value = 2.9 × 10^–9^).

**Fig. 8:**
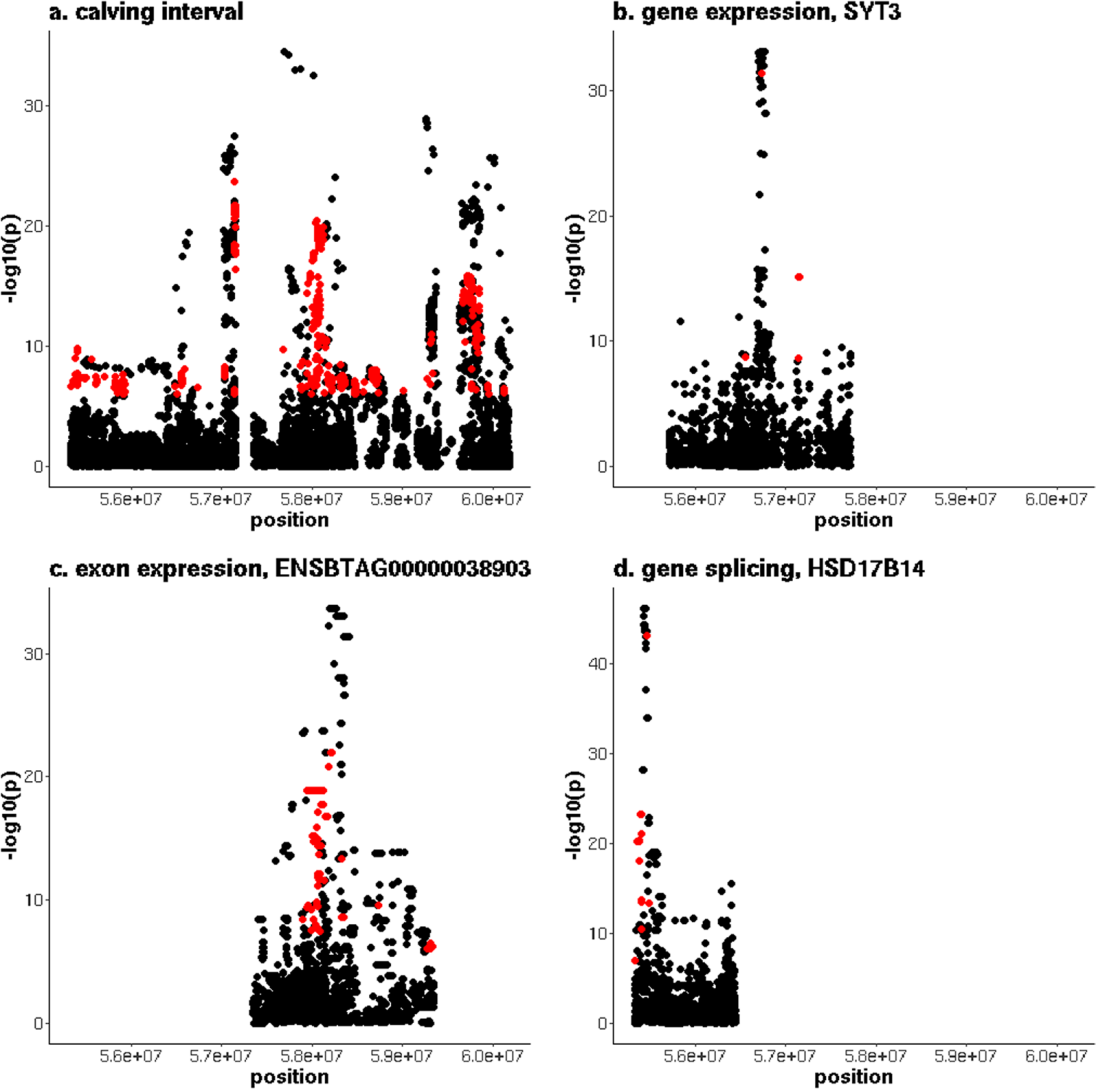
Overlap between a QTL for calving interval on chromosome 18 and multiple types of eQTL. **a** y-axis = − log10(p) for the meta-analysis of calving interval (CI), **b** y-axis = − log10(p) for *SYT3* expression, **c** y-axis = − log10(p) for exon expression of exon of *ENSBTAG00000038903* located between 58,342,620 and 58,343,553 bp, and (**d**) y-axis = − log10(p) for the splice region between 55,443,019 and 55,445,791 bp in in *HSD17B14*, red indicates variants that are significant (p ≤ 10^–6^) for both CI and expression phenotype. Expression associations were only tested for variants within 1 Mb of the gene/exon/splice region; the gaps on the gene/exon/splice graphs fall outside these boundaries

Chromosome 18 contained the QTL with the strongest association with CI, located between 55,331,602 and 60,178,330 bp. This QTL overlapped with significant geQTL, eeQTL and sQTL (Fig. 8). The QTL region contained 545 variants that were significantly associated with both CI and a geQTL (see Additional file 7: Table S6). The most significant geQTL (p = 3.7 × 10^–32^) was associated with the expression of the *synaptotagmin 3* (*SYT3*) gene. Many variants were associated with the expression of multiple genes (see Additional file 8: Table S7). Forty-two variants were located between 55,816,529 and 55,937,694 bp, associated with the expression of both the *Fms related receptor tyrosine kinase 3 ligand* (*FLT3LG*) and *solute carrier family 6 member 16* (*SLC6A16)* genes. For all these 42 variants, the alleles that were associated with increased CI were associated with increased expression of *FLT3LG* and decreased expression of *SLC6A16*. Another group of 53 variants, located between 56,556,940 and 57,152,300 bp, were associated with both the expression of *SYT3* and *ENSBTAG00000037537*; 43 and two of these variants were also associated with the expression of the *DNA polymerase delta 1, catalytic subunit* (*POLD1*) and *chromosome 18 C19orf81 homolog* (*C18H19orf81*) genes, respectively. For these variants, the alleles that were associated with increased CI were associated with reduced expression of *SYT3, ENSBTAG00000037537, POLD1* and *C18H19orf81*. The largest group of genes for which an overlap of eQTL was detected contained seven genes (*zinc finger protein 350* (*ZNF350*), *ENSBTAG00000050420, ENSBTAG00000019227, ENSBTAG00000038903, ENSBTAG00000033523, ENSBTAG00000053131* and *ENSBTAG00000017651*) for which we detected 277 variants, located between 57,873,772 and 58,735,393 bp, that were each associated with the expression of at least two of the genes in this group. The largest overlap was found between *ENSBTAG00000038903* and *ENSBTAG00000033523*, with 273 variants significantly associated with the expression of both genes. For variants associated with the expression of genes in this group, the alleles that were associated with increased CI increased the expression of *ENSBTAG00000038903*, *ENSBTAG00000033523* and *ENSBTAG00000053131*, reduced the expression of *ZNF350* and *ENSBTAG00000017651*, while the direction of effect was not consistent for *ENSBTAG00000050420* and *ENSBTAG00000019227* (i.e., for some the variants, the alleles that increased CI increased gene expression, while for others the alleles that increased CI decreased gene expression). The last group of genes with overlapping geQTL included *ENSBTAG00000047761* and *ENSBTAG00000015899*. Thirteen variants were associated with the expression of both these genes, located between 58,665,839 and 58,695,910 bp. For these variants, the alleles that were associated with increased CI increased the expression of *ENSBTAG00000047761* and decreased the expression of *ENSBTAG00000015899*. The same QTL region included 429 variants associated with both CI and the expression of exons of the *FLT3LG*, *C18H19orf81*, *ENSBTAG00000037537*, *ZNF350*, *ENSBTAG00000050420*, *ENSBTAG00000019227*, *ENSBTAG00000038903*, *ENSBTAG00000033523* and *ENSBTAG00000017651* genes. The most significant eeQTL (p = 9.5 × 10^–23^) was associated with the exon between 58,342,620 and 58,343,553 bp in *ENSBTAG00000038903*. Several of the variants associated with the expression of this exon were located in the same CNV, between 58,172,482 and 58,285,235 bp [43], that encompassed five genes (*ENSBTAG00000049460*, *ENSBTAG00000050064*, *ENSBTAG00000050488*, *ENSBTAG00000054038* and *ENSBTAG00000054547*). Six hundred and ninety variants were associated with both CI and the expression of at least one of the 243 splice regions. The most significant sQTL (p = 5.7 × 10^–44^) was associated with a splice region in the *hydroxysteroid 17-beta dehydrogenase 14* (*HSD17B14*) gene between 55,443,019 and 55,445,791 bp.

## Discussion

We identified multiple QTL associated with fertility in dairy cattle that overlap with eQTL. Variants that are significantly associated with CI are highly enriched for variants associated with gene expression, exon expression, gene splicing and ASE. This enrichment is substantially larger than previously reported for production and fertility traits in Australian dairy cattle [44]. The increased enrichment may be explained by the larger number of samples with expression data used in our study, the difference in the tissues used, and the difference in the populations analysed.

### Differential expression

We identified 671 genes that were differentially expressed between the POS and NEG cows. While we also detected geQTL for several of these genes, none of these geQTL overlapped with QTL detected for CI. This may be because CI does not capture all the genetic variance associated with fertility, and the POS and NEG cows were selected based on traits other than CI. A number of the differentially expressed genes detected in our study have previously been reported in similar studies. For example, 28 of the differentially expressed genes were reported as significantly differentially expressed between the endometrium of fertile and subfertile New Zealand dairy cows during early pregnancy [45]. However, only 13 of these 28 had a consistent direction of effect (i.e., upregulated in the POS cows in our study, and upregulated in the fertile cows studied by Walker et al. [45]). The largest overlap was found with Moran et al. [46]: we detected 96 differentially expressed genes that were also differentially expressed in liver or muscle tissue of Irish Holstein cows with high or low genetic merit for fertility, at three different time points (late pregnancy, early lactation and mid-lactation). The overlap between our study and Moran et al. [46] includes several of the genes with the largest fold changes in our analyses: *CCDC196*, *CPXM2*, *ISG12(B)*, *CHI3L2*, *ATP6V1C2* and *MS4A3*. However, similar to the overlap with Walker et al. [45], the direction of the fold change did not always correspond between our results and those of Moran et al. [46]. We found a greater concordance with Moran et al. [46] for the genes that were differentially expressed in liver tissue and during early lactation (e.g., the same tissue and closest time period in our study), than in the muscle tissue or during the other time periods. Moran et al. [46] reported several genes for which the direction of fold change differed between the three time periods. For example, the *ISG12(B)* gene was upregulated in the POS NZ cows and in the liver samples from Moran et al. [46] during early lactation, but downregulated during late pregnancy and mid lactation. The two genes with the largest fold change in our study, *CCDC196* and *CPXM2*, were both downregulated in the POS NZ cows, but not significantly differentially expressed during early lactation and upregulated during late pregnancy and mid lactation in the study of Moran et al. [46]. The *CCDC196* gene has also been reported to be upregulated in endometrial samples of beef heifers after mating [47]. The *CPXM2* gene was found to be downregulated in the endometrium of non-lactating Japanese Black cows that failed to conceive after at least three inseminations, e.g., cows with reduced fertility [48], and in dairy sheep, its expression increased substantially during lactation [49]. Hence, while several studies report the same genes as candidate genes for various fertility traits, their impact on fertility appears to depend on pregnancy stage. Hence, to better understand how the differentially expressed genes impact fertility, further studies that would include samples taken throughout lactation and in tissues directly related to reproduction are necessary.

### Overlap between QTL for CI and eQTL

The overlap between QTL for CI and eQTL identified several candidate genes for fertility. The *GYS2* gene on chromosome 5 has previously been reported to be upregulated in dairy cows with imbalanced metabolic profiles in early lactation [50]. In humans, *GYS2* has been associated with glycogen storage disease type 0 [51], obesity and polycystic ovary syndrome [52]. The QTL region, including a second candidate gene, *TIGAR*, on chromosome 5, has previously been reported to be associated with age at puberty in New Zealand Holstein-Friesidan dairy cattle [53], and a range of traits in various cattle populations, including adaptation to high altitude, response to hypoxia, body size and stature in Swedish dairy cattle [54], milking speed in French Holstein cows [55], multiple body weight traits in Hereford cattle [56] and metabolic body weight in Holstein cows [57]. Furthermore, Liu et al. [58] reported colocalisation of an eQTL in *TIGAR* expression in muscle and loci identified by GWAS associated with strength in cattle. The QTL region detected on chromosome 6, associated with the expression of *GC*, is a well-known pleiotropic QTL in dairy cattle, associated with production traits, mastitis resistance, mammary gland morphology and fertility [59–62]. Recently, Lee et al. [12] provided evidence that the causal mutation underlying this QTL in Dutch Holstein Friesian cattle is likely to be a ~ 12 kb multi-allelic CNV. Five of the six variants that tagged the CNV in the study by Lee et al. [12] were present in our study, and all five were significantly associated with both CI and *GC* expression. A QTL region on chromosome 15 contained multiple genes from the olfactory receptor gene family. Several of the variants associated with this QTL were located within a CNV. Both in humans [63] and cattle [64], the olfactory receptor gene family region is known to contain a large number of CNV. Other genes associated with this QTL included *ARHGAP1*, for which the endometrial expression has been found to increase in beef cattle during early pregnancy [65], and *CREB3L1*, which has been associated with heat tolerance in Holstein cows [66]. On chromosome 18, the most significant QTL region is a well-known QTL for fertility [67–69], and contains many CNV [43]. This QTL overlapped with a large number of eQTL, and many of the genes associated with the eQTL are novel genes with little to no functional annotation available. The most significant sQTL was associated with a splice region of the *HSD17B14* gene, which has functions that include the oxidisation of oestradiol and testosterone and is expressed in the breast, ovary and testis in humans [70]. Interestingly, this gene is a member of the same gene family as *HSD17B12* (chromosome 15) that was associated with submission rate by day 21 of the breeding season in NZ cows in a previous study [19]. However, the missense mutation in *HSD17B12* that was detected by Juengel et al. [19] was not associated with CI in AUS (p = 0.04) and we did not detect an eQTL associated with this variant. The lack of association with CI may be explained by the difference in traits: while both relate to female fertility, they are different traits and are not necessarily expected to share all QTL.

Several QTL for CI that overlapped with eQTL were detected in regions that contained one or more structural variants such as CNV [42, 43]. In particular, the region on chromosome 18 that has the most significant QTL for CI contains multiple structural variants [43, 71]. Hence, it is likely that the causal variants for some of the QTL in our study are structural variants, such as the QTL associated with *GC* expression [12]. However, because we imputed only SNPs and small INDELs from a short read sequence reference set, our dataset does not include the structural variants that may be the causal mutations. This could explain why for some QTL regions, none of the variants present had a consistent effect in the AUS and NZ data because they may tag the absent causal mutation and the LD phase may differ. Further fine mapping of the QTL regions spanning CNV, such as on chromosome 18, may be required using long read sequencing to identify the causal mutation underlying the QTL.

While we found significant enrichment for almost all the significance thresholds tested, the enrichment fold depended on the significance threshold used. The higher enrichment of geQTL and eeQTL using more stringent thresholds are likely highly influenced by the most significant QTL for CI on chromosomes 6 and 18. Both these QTL overlapped with eQTL, and because our enrichment analyses did not account for LD, many variants contributing to the enrichment analyses are likely associated with the same causal mutation. Hence, while approximately 50% of significant variants for CI were also associated with gene expression, this does not mean that 50% of all causal mutations of CI were expected to also be causal mutations of geQTL. Because we used different datasets for the GWAS for CI and the eQTL analyses, we did not attempt colocalisation, as it would be challenging to fit LD estimates appropriate for both populations. Therefore, further analyses are required to confirm whether the identified overlapping regions between QTL and eQTL are caused by the same or different causal mutations. While this was a disadvantage of using different datasets for the GWAS for CI and the eQTL analyses, using these different datasets did allow us to combine the largest (e.g., most power for QTL detection) available dataset for a fertility trait with the NZ selection experiment, and detect regions that are likely to impact fertility in both AUS and NZ dairy cattle populations.

## Conclusions

We combined a powerful meta-analysis for fertility with gene expression results in cattle that were divergently selected for high and low fertility to identify putative candidate genes associated with fertility traits. Variants that were significantly associated with CI were highly enriched for geQTL, eeQTL, sQTL and aseQTL. We detected 671 genes that were differentially expressed between POS and NEG cows, with the largest fold change detected for the *CCDC196* gene on chromosome 10. Several QTL detected for CI overlapped with eQTL, providing candidate genes for fertility in dairy cattle. Multiple QTL regions were located in regions with large numbers of CNV. To identify the causal mutations underlying these QTL, long read sequencing may be required.

### Supplementary Information

Additional file 1: Table S1. Title: Descriptive statistics for the New Zealand fertility traits. Description: Trait, description and unit for the New Zealand fertility phenotypes measured as heifer (sheet 1) and during the lactation 1 (sheet 2) and lactation 2 (sheet 3). For each trait, the number of records (n), mean and standard deviation (sd) are given for both the low- and high-fertility cows.

Additional file 2: Figure S1. Title: Comparison of the direction of effect in different analyses. Description: An example of when an allele has a concordant or non-concordant direction of effect in different parts of the analyses.

Additional file 3: Table S2. Title: Descriptive statistics of RNA sequencing.

Additional file 4: Table S3. Title: False discovery rates and enrichment analyses for different significance thresholds. Description: False discovery rates for the detection of QTL associated with calving interval, and false discovery rates and enrichment analyses for gene expression QTL, exon expression QTL, gene splicing QTL and allele specific expression QTL, using significance thresholds of 10^-4^, 10^-6^, and 10^-8^.

Additional file 5: Table S4. Title: Percentage of variants with a direction of effect on fertility that is consistent in Australia and New Zealand, for New Zealand traits measured as heifer (sheet 1) and during lactation 2 (sheet 2). Description: Trait = New Zealand fertility phenotype, agepub = age at puberty, co3wk/co6wk/co9wk/co12wk = pregnancy rate at 3/6/9/12 weeks, ppai = Prolonged postpartum anovulatory intervals, ppaicens = ppai censored, preg = pregnancy rate, sm3wk/sm6wk = submission rate at 3/6 weeks, tstai1 = time from planned start of mating to 1st AI, tstconc = time from planned start of mating to conception, ttai1 = time from calving to 1st AI, ttconc = time to conception, conccens = ttconc censored, ALL = all variants included in the study, CI = variants significantly associated (p ≤ 10^-6^) with calving interval in Australia, CI+GE/CI+EE/CI+SPLICE/CI+ASE = variants significantly associated (p ≤ 10^-6^) with calving interval in Australia and gene expression/exon expression/gene splicing/allele specific expression in New Zealand.

Additional file 6: Table S5. Title: List of genes significantly differentially expressed between high- and low-fertility cows. Description: genes with a false discovery rate (FDR) < 0.05 were declared significant. ENSEMBL_gene_ID = Ensembl stable gene identifier, gene_name = gene name, chr = chromosome, start = start of gene in base pair (bp), end = end of gene in bp, logFC = logarithm of fold change, topGE = most significant variant in the gene expression GWAS (if there were more than one variant with the smallest p-value, we selected the variant that had the smallest p-value in the meta-analysis for calving interval), pGE = p-value in the gene expression GWAS, effectGE = effect estimated in the gene expression GWAS, pCI = p-value in the meta-analysis for calving interval, effectCI = Z-score estimated in the meta-analysis for calving interval).

Additional file 7: Table S6. Title: Details on the variants that were significantly associated with calving interval and expression phenotypes. Description: Details are provided for each of the examples highlighted in the Results section; each tab corresponds to one example of overlap between a QTL for calving interval and significant variants associated with one of the expression phenotypes. This excel table contains 12 sheets, respectively for an sQTL chromosome 5, a geQTL chromosome 5, a geQTL on chromosome 6, an eeQTL chromosome 6, an aseQTL on chromosome 6, a geQTL on chromosome 15, an eeQTL on chromosome 15, an sQTL on chromosome 15, an aseQTL on chromosome 15, a geQTL on chromosome 18, an eeQTL on chromosome 18, an eeQTL on chromosome 18, and an sQTL on chromosome 18.

Additional file 8: Table S7. Title: Genes associated with geQTL on chromosome 18. Description: The diagonal shows the number of variants that were significantly associated with the expression of the gene and with CI, the off-diagonal shows how many of these variants overlapped between a pair of genes.

## Data Availability

Data may be available upon request to DataGene and DairyNZ.

## References

[1] Berry DP, Wall E, Pryce JE (2014). Genetics and genomics of reproductive performance in dairy and beef cattle. Animal.

[2] Lucy MC (2019). Symposium review: Selection for fertility in the modern dairy cow—current status and future direction for genetic selection. J Dairy Sci.

[3] Cole JB, VanRaden PM (2018). Symposium review: possibilities in an age of genomics: the future of selection indices. J Dairy Sci.

[4] Ma L, Cole JB, Da Y, VanRaden PM (2019). Symposium review: genetics, genome-wide association study, and genetic improvement of dairy fertility traits. J Dairy Sci.

[5] Workie ZW, Gibson JP, van der Werf JHJ (2019). Age at culling and reasons of culling in Australian dairy cows. Proc Assoc Advmt Anim Breed Genet.

[6] van den Berg I, Boichard D, Lund MS (2016). Sequence variants selected from a multi-breed GWAS can improve the reliability of genomic predictions in dairy cattle. Genet Sel Evol.

[7] Brøndum RF, Su G, Janss L, Sahana G, Guldbrandtsen B, Boichard D (2015). Quantitative trait loci markers derived from whole genome sequence data increases the reliability of genomic prediction. J Dairy Sci.

[8] van den Berg I, Boichard D, Guldbrandtsen B, Lund MS (2016). Using sequence variants in linkage disequilibrium with causative mutations to improve across-breed prediction in dairy cattle: a simulation study. BG3 (Gethesda)..

[9] Cai Z, Guldbrandtsen B, Lund MS, Sahana G (2019). Prioritizing candidate genes for fertility in dairy cows using gene-based analysis, functional annotation and differential gene expression. BMC Genomics.

[10] Marete AG, Guldbrandtsen B, Lund MS, Fritz S, Sahana G, Boichard D (2018). A meta-analysis including pre-selected sequence variants associated with seven traits in three French dairy cattle populations. Front Genet.

[11] Chen S-Y, Schenkel FS, Melo ALP, Oliveira HR, Pedrosa VB, Araujo AC (2022). Identifying pleiotropic variants and candidate genes for fertility and reproduction traits in Holstein cattle via association studies based on imputed whole-genome sequence genotypes. BMC Genomics.

[12] Lee Y-L, Takeda H, Costa Monteiro Moreira G, Karim L, Mullaart E, Coppieters W (2021). A 12 kb multi-allelic copy number variation encompassing a GC gene enhancer is associated with mastitis resistance in dairy cattle. PLoS Genet.

[13] Littlejohn MD, Tiplady K, Fink TA, Lehnert K, Lopdell T, Johnson T (2016). Sequence-based association analysis reveals an MGST1 eQTL with pleiotropic effects on bovine milk composition. Sci Rep.

[14] Xiang R, Fang L, Liu S, Macleod IM, Liu Z, Breen EJ (2023). Gene expression and RNA splicing explain large proportions of the heritability for complex traits in cattle. Cell Genomics.

[15] Meier S, McNaughton L, Handcock R, Amer P, Beatson P, Bryant J (2021). Heifers with positive genetic merit for fertility traits reach puberty earlier and have a greater pregnancy rate than heifers with negative genetic merit for fertility traits. J Dairy Sci.

[16] Meier S, Kuhn-Sherlock B, Amer P, Roche J, Burke C (2021). Positive genetic merit for fertility traits is associated with superior reproductive performance in pasture-based dairy cows with seasonal calving. J Dairy Sci.

[17] Grala TM, Kuhn-Sherlock B, Crookenden MA, Walker CG, Roche JR, Price MD (2022). Adaptive immune response ranking is associated with reproductive phenotypes in grazing dairy cows divergent in genetic merit for fertility traits. J Dairy Sci.

[18] Grala TM, Kuhn-Sherlock B, Roche JR, Jordan OM, Phyn CVC, Burke CR (2022). Changes in plasma electrolytes, minerals, and hepatic markers of health across the transition period in dairy cows divergent in genetic merit for fertility traits and postpartum anovulatory intervals. J Dairy Sci.

[19] Juengel J, Mosaad E, Mitchell M, Phyn C, French M, Meenken E (2022). Relationships between prostaglandin concentrations, a single nucleotide polymorphism in HSD17B12, and reproductive performance in dairy cows. J Dairy Sci.

[20] Haile-Mariam M, Bowman PJ, Pryce JE (2013). Genetic analyses of fertility and predictor traits in Holstein herds with low and high mean calving intervals and in Jersey herds. J Dairy Sci.

[21] VanRaden PM, Wiggans GR (1991). Derivation, calculation, and use of national animal model information. J Dairy Sci.

[22] Rosen BD, Bickhart DM, Schnabel RD, Koren S, Elsik CG, Tseng E (2020). De novo assembly of the cattle reference genome with single-molecule sequencing. Gigascience..

[23] Sargolzaei M, Chesnais JP, Schenkel FS (2014). A new approach for efficient genotype imputation using information from relatives. BMC Genomics.

[24] Loh P-R, Palamara PF, Price AL (2016). Fast and accurate long-range phasing in a UK Biobank cohort. Nat Genet.

[25] Das S, Forer L, Schönherr S, Sidore C, Locke AE, Kwong A (2016). Next-generation genotype imputation service and methods. Nat Genet.

[26] Daetwyler HD, Capitan A, Pausch H, Stothard P, van Binsbergen R, Brøndum RF (2014). Whole-genome sequencing of 234 bulls facilitates mapping of monogenic and complex traits in cattle. Nat Genet.

[27] Hayes BJ, Daetwyler HD (2019). 1000 bull genomes project to map simple and complex genetic traits in cattle: applications and outcomes. Annu Rev Anim Biosci.

[28] Andrews S. FastQC: a quality control tool for high throughput sequence data. 2010. https://www.bioinformatics.babraham.ac.uk/projects/fastqc/ Accessed 17 Apr 2024.

[29] Robinson AJ, Ross EM (2019). QuAdTrim: overcoming computational bottlenecks in sequence quality control. bioRxiv..

[30] Dobin A, Davis CA, Schlesinger F, Drenkow J, Zaleski C, Jha S (2013). STAR: ultrafast universal RNA-seq aligner. Bioinformatics.

[31] Liao Y, Smyth GK, Shi W (2014). featureCounts: an efficient general purpose program for assigning sequence reads to genomic features. Bioinformatics.

[32] Robinson MD, Oshlack A (2010). A scaling normalization method for differential expression analysis of RNA-seq data. Genome Biol.

[33] Robinson MD, McCarthy DJ, Smyth GK (2010). edgeR: a Bioconductor package for differential expression analysis of digital gene expression data. Bioinformatics.

[34] Li YI, Knowles DA, Humphrey J, Barbeira AN, Dickinson SP, Im HK (2018). Annotation-free quantification of RNA splicing using LeafCutter. Nat Genet.

[35] Van der Auwera GA, O’Connor BD. Genomics in the cloud: using Docker, GATK, and WDL in Terra. Sebastopol: O’Reilly Media; 2020.

[36] Yang J, Lee SH, Goddard ME, Visscher PM (2011). GCTA: a tool for genome-wide complex trait analysis. Am J Hum Genet.

[37] Yang J, Benyamin B, McEvoy BP, Gordon S, Henders AK, Nyholt DR (2010). Common SNPs explain a large proportion of the heritability for human height. Nat Genet.

[38] Willer CJ, Li Y, Abecasis GR (2010). METAL: fast and efficient meta-analysis of genomewide association scans. Bioinformatics.

[39] Benjamini Y, Hochberg Y (1995). Controlling the false discovery rate: a practical and powerful approach to multiple testing. J R Stat Soc.

[40] Prowse-Wilkins CP, Lopdell TJ, Xiang R, Vander Jagt CJ, Littlejohn MD, Chamberlain AJ (2022). Genetic variation in histone modifications and gene expression identifies regulatory variants in the mammary gland of cattle. BMC Genomics.

[41] R Core Team. R: A language and environment for statistical computing. Vienna: R Foundation for Statistical Computing; 2015.

[42] Boussaha M, Esquerré D, Barbieri J, Djari A, Pinton A, Letaief R (2015). Genome-wide study of structural variants in bovine Holstein, Montbéliarde and Normande dairy breeds. PLoS ONE.

[43] Bickhart DM, Hou Y, Schroeder SG, Alkan C, Cardone MF, Matukumalli LK (2012). Copy number variation of individual cattle genomes using next-generation sequencing. Genome Res.

[44] van den Berg I, Hayes BJ, Chamberlain AJ, Goddard ME (2019). Overlap between eQTL and QTL associated with production traits and fertility in dairy cattle. BMC Genomics.

[45] Walker CG, Littlejohn MD, Mitchell MD, Roche JR, Meier S (2012). Endometrial gene expression during early pregnancy differs between fertile and subfertile dairy cow strains. Physiol Genomics.

[46] Moran B, Cummins SB, Creevey CJ, Butler ST (2016). Transcriptomics of liver and muscle in Holstein cows genetically divergent for fertility highlight differences in nutrient partitioning and inflammation processes. BMC Genomics.

[47] Recuero S, Sánchez JM, Mateo-Otero Y, Bagés-Arnal S, McDonald M, Behura SK (2020). Mating to intact, but not vasectomized, males elicits changes in the endometrial transcriptome: insights from the bovine model. Front Cell Dev Biol.

[48] Hayashi K-G, Hosoe M, Kizaki K, Fujii S, Kanahara H, Takahashi T (2017). Differential gene expression profiling of endometrium during the mid-luteal phase of the estrous cycle between a repeat breeder (RB) and non-RB cows. Reprod Biol Endocrinol.

[49] Suárez-Vega A, Gutiérrez-Gil B, Klopp C, Robert-Granie C, Tosser-Klopp G, Arranz JJ (2015). Characterization and comparative analysis of the milk transcriptome in two dairy sheep breeds using RNA sequencing. Sci Rep.

[50] Wathes DC, Cheng Z, Salavati M, Buggiotti L, Takeda H, Tang L (2021). Relationships between metabolic profiles and gene expression in liver and leukocytes of dairy cows in early lactation. J Dairy Sci.

[51] Soggia AP, Correa-Giannella ML, Fortes MAH, Luna AMC, Pereira MAA (2010). A novel mutation in the glycogen synthase 2 gene in a child with glycogen storage disease type 0. BMC Med Genet.

[52] Hwang J-Y, Lee E-J, Go MJ, Sung Y-A, Lee HJ, Kwak SH (2012). Genome-wide association study identifies GYS2 as a novel genetic factor for polycystic ovary syndrome through obesity-related condition. J Hum Genet.

[53] Stephen MA, Burke CR, Steele N, Pryce JE, Meier S, Amer PR, et al. Genome-Wide Association Study of age at puberty and its (co)variances with fertility and stature in growing and lactating Holstein-Friesian dairy cattle. J Dairy Sci. 2023. 10.3168/jds.2023-23963 38135043

[54] Ghoreishifar SM, Eriksson S, Johansson AM, Khansefid M, Moghaddaszadeh-Ahrabi S, Parna N (2020). Signatures of selection reveal candidate genes involved in economic traits and cold acclimation in five Swedish cattle breeds. Genet Sel Evol.

[55] Marete A, Sahana G, Fritz S, Lefebvre R, Barbat A, Lund MS (2018). Genome-wide association study for milking speed in French Holstein cows. J Dairy Sci.

[56] Saatchi M, Schnabel RD, Taylor JF, Garrick DJ (2014). Large-effect pleiotropic or closely linked QTL segregate within and across ten US cattle breeds. BMC Genomics.

[57] Hardie LC, VandeHaar MJ, Tempelman RJ, Weigel KA, Armentano LE, Wiggans GR (2017). The genetic and biological basis of feed efficiency in mid-lactation Holstein dairy cows. J Dairy Sci.

[58] Liu S, Gao Y, Canela-Xandri O, Wang S, Yu Y, Cai W (2022). A multi-tissue atlas of regulatory variants in cattle. Nat Genet.

[59] Sodeland M, Grove H, Kent M, Taylor S, Svendsen M, Hayes BJ (2011). Molecular characterization of a long range haplotype affecting protein yield and mastitis susceptibility in Norwegian Red cattle. BMC Genet.

[60] Pausch H, Emmerling R, Schwarzenbacher H, Fries R (2016). A multi-trait meta-analysis with imputed sequence variants reveals twelve QTL for mammary gland morphology in Fleckvieh cattle. Genet Sel Evol.

[61] Jiang J, Ma L, Prakapenka D, VanRaden PM, Cole JB, Da Y (2019). A large-scale genome-wide association study in US Holstein cattle. Front Genet.

[62] Tribout T, Croiseau P, Lefebvre R, Barbat A, Boussaha M, Fritz S (2020). Confirmed effects of candidate variants for milk production, udder health, and udder morphology in dairy cattle. Genet Sel Evol.

[63] Young JM, Endicott RM, Parghi SS, Walker M, Kidd JM, Trask BJ (2008). Extensive copy-number variation of the human olfactory receptor gene family. Am J Hum Genet.

[64] Lee K, Nguyen DT, Choi M, Cha S-Y, Kim J-H, Dadi H (2013). Analysis of cattle olfactory subgenome: the first detail study on the characteristics of the complete olfactory receptor repertoire of a ruminant. BMC Genomics.

[65] Forde N, Duffy GB, McGettigan PA, Browne JA, Mehta JP, Kelly AK (2012). Evidence for an early endometrial response to pregnancy in cattle: both dependent upon and independent of interferon tau. Physiol Genomics.

[66] Sigdel A, Abdollahi-Arpanahi R, Aguilar I, Peñagaricano F (2019). Whole genome mapping reveals novel genes and pathways involved in milk production under heat stress in US Holstein cows. Front Genet.

[67] Cole JB, VanRaden PM, O’Connell JR, Van Tassell CP, Sonstegard TS, Schnabel RD (2009). Distribution and location of genetic effects for dairy traits. J Dairy Sci.

[68] Sahana G, Guldbrandtsen B, Lund MS (2011). Genome-wide association study for calving traits in Danish and Swedish Holstein cattle. J Dairy Sci.

[69] Abo-Ismail MK, Brito LF, Miller SP, Sargolzaei M, Grossi DA, Moore SS (2017). Genome-wide association studies and genomic prediction of breeding values for calving performance and body conformation traits in Holstein cattle. Genet Sel Evol.

[70] Sivik T, Vikingsson S, Gréen H, Jansson A (2012). Expression patterns of 17β-hydroxysteroid dehydrogenase 14 in human tissues. Horm Metab Res.

[71] Dachs N, Upadhyay M, Hannemann E, Hauser A, Krebs S, Seichter D (2023). Quantitative trait locus for calving traits on Bos taurus autosome 18 in Holstein cattle is embedded in a complex genomic region. J Dairy Sci.

