# Supplementary figures and images for: Using expression data to fine map QTL associated with fertility in dairy cattle
Source: Genet Sel Evol. 2024 Jun 6;56:42. doi: 10.1186/s12711-024-00912-8 (PMC11154999; doi:10.1186/s12711-024-00912-8)

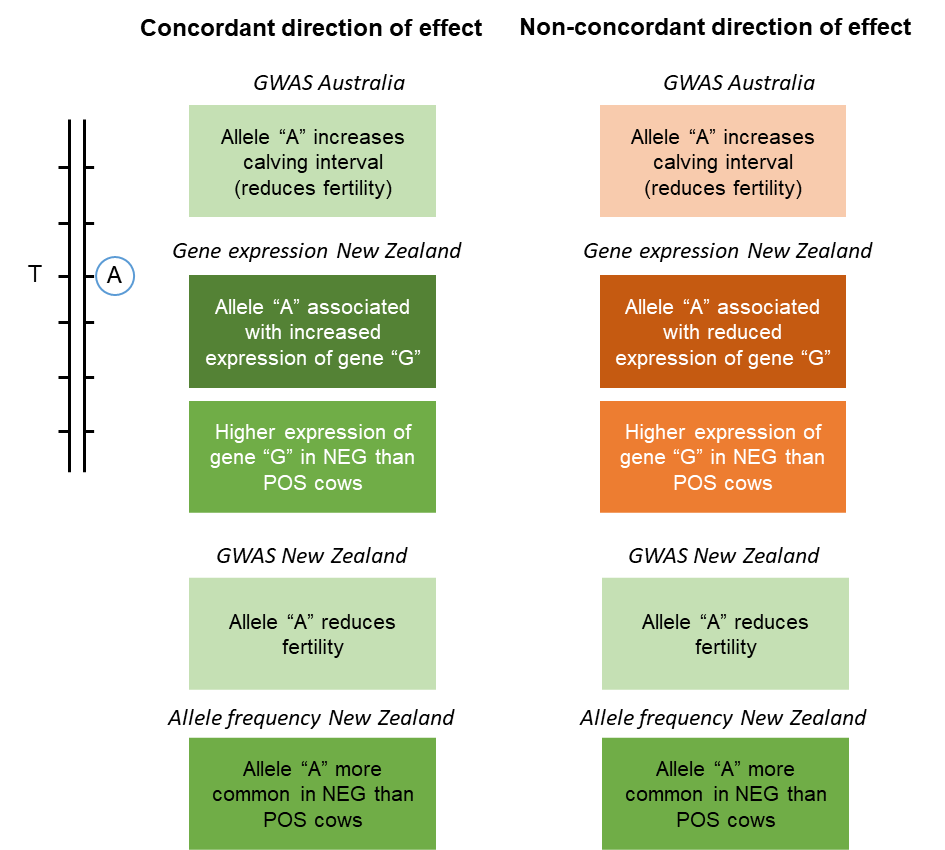

Supplement: Supplementary file 2 — Additional file 2: Figure S1. Title: Comparison of the direction of effect in different analyses. Description: An example of when an allele has a concordant or non-concordant direction of effect in different parts of the analyses. [file 12711_2024_912_MOESM2_ESM.png]
