# Supplementary material for: Using expression data to fine map QTL associated with fertility in dairy cattle
Source: Genet Sel Evol. 2024 Jun 6;56:42. doi: 10.1186/s12711-024-00912-8 (PMC11154999; doi:10.1186/s12711-024-00912-8)
Supplement: Supplementary file 3 — Additional file 3: Table S2. Title: Descriptive statistics of RNA sequencing. [file 12711_2024_912_MOESM3_ESM.docx]

| Number of cows | 393 |
| --- | --- |
| Average number of read pairs per sample | 24,513,204 |
| Minimum number of read pairs per sample | 21 |
| Maximum number of read pairs per sample | 183,628,008 |
| Standard deviation of number of read pairs | 13,725,227 |
| Average number of uniquely aligned pairs | 21,889,472 |
| Average % uniquely aligned pairs | 89.32% |
| Average % multi aligned pairs | 2.22% |
| Average % unmapped mismatches | 0.00% |
| Average % unmapped short | 8.36% |
| Average % unmapped other | 0.03% |
| Number of cows after QC* | 365 |

* Samples with > 10^-7^ read pairs and % uniquely aligned pairs > 60
